# Homozygous Familial Hypercholesterolemia Treatment: New Developments

**DOI:** 10.1007/s11883-024-01269-5

**Published:** 2025-01-03

**Authors:** Dirk J. Blom, A. David Marais, Frederick J. Raal

**Affiliations:** 1 Division of Lipidology and Cape Heart Institute, Department of Medicine, University of Cape Town, Cape Town, South Africa; 2 Division of Chemical Pathology, Department of Pathology, University of Cape Town, Cape Town, South Africa; 3 Carbohydrate and Lipid Metabolism Research Unit, Department of Medicine, University of the Witwatersrand, Johannesburg, South Africa

**Keywords:** Homozygous familial hypercholesterolemia, Proprotein convertase subtilisin kexin type 3, Angiopoietin like factor 3, Microsomal, Triglyceride transfer protein, Gene editing

## Abstract

**Purpose of review:**

Homozygous familial hypercholesterolaemia (HoFH) is characterized by marked elevation of low-density lipoprotein cholesterol (LDLC) and premature atherosclerotic cardiovascular disease. This is a review of novel pharmacological therapies to lower LDLC in patients with HoFH.

**Recent findings:**

Novel therapies can be broadly divided by whether their efficacy is dependent or independent of residual low-density lipoprotein receptor (LDLR) function. Novel LDLR dependent therapies that reduce proprotein subtilisin kexin type 9 levels include monoclonal antibodies (alirocumab and evolocumab) and a small inhibitory RNA (inclisiran). LDLC reductions are highly variable and depend on residual LDLR function. Microsomal triglyceride inhibitors (lomitapide) and therapies that reduce angiopoietin like factor 3 (evinacumab and zodasiran) both reduce LDLC by approximately 50%, irrespective of residual LDLR function.

**Summary:**

Most patients with HoFH require multiple therapies to achieve LDLC targets. Better LDLC control with LDLR independent therapies is likely to improve the outlook for patients with HoFH while at the same time reducing the need for other therapies such as apheresis or hepatic transplantation.

## Introduction

Clinically, the term homozygous familial hypercholesterolemia (HoFH) describes a phenotype characterized by extreme elevation of low-density lipoprotein cholesterol (LDLC) (classically > 13 mmol/L in the untreated state), presence of cutaneous and tendinous xanthomata from a young age, markedly premature cardiovascular disease, supravalvular aortic stenosis and inherited hypercholesterolemia in both parents in most cases [1]. More recent studies have identified patients with genetically confirmed HoFH who have biochemically and clinically milder phenotypes with lower untreated LDLC levels than the classic description of HoFH [2]. Genetically HoFH is a complex disorder defined by the presence of bi-allelic pathogenic variants in one of four genes associated with FH. Pathogenic variants in the *LDLR*, *ApoB* and *PCSK9* genes are inherited in a semi-dominant fashion and cause heterozygous familial hypercholesterolemia (HeFH) when present on a single allele, while pathogenic variants in the *LDLRAP1* protein confer an autosomal recessive inheritance pattern and only cause HoFH when present on both alleles [1, 3]. Bi-allelic pathogenic variants in the LDLR are found in 85–90% of patients with HoFH. Other patients either carry bi-allelic pathogenic variants in one of the other genes associated with FH, or more rarely have pathogenic variants in two (or more) FH-associated genes (digenic inheritance). Physiologically HoFH is characterized by marked LDL receptor (LDLR) dysfunction. Functionally pathogenic variants in the *LDLR* gene are often characterized by their residual activity with null variants having less than 2% residual activity and defective variants > 2% activity. Ideally, residual functional activity should be determined by functional cell-based studies, but such data is available only for a minority of pathogenic variants and residual functional activity is often determined by predictive algorithms or remains unknown for many pathogenic variants [4]. Residual LDLR activity is an important determinant of response to medications that upregulate hepatic LDLR expression. Patients with a null/null status generally respond poorly, or not at all, to medications whose predominant mechanism of action is LDLR upregulation [1].

The treatment of HoFH focuses on early diagnosis and early and aggressive reduction of LDLC. The most recent consensus statement from the European Atherosclerosis Society (EAS) proposes that adult patients with HoFH be treated to the high-risk LDLC target of < 1.8 mmol/L unless they have established atherosclerotic cardiovascular disease (ASCVD) or other risk factors when they should be treated to the very-high risk LDLC target of < 1.4 mmol/L. HoFH patients under 18 years should be treated to an LDLC target of < 3.0 mmol/L unless there is clinical or imaging evidence of ASCVD when the target should be lowered [1]. Given the clinical severity of HoFH and magnitude of LDLC elevation it would be unethical to conduct any cardiovascular outcome studies in this population using placebo or even less intensive versus more intensive therapy, as only a minority of patients achieve LDLC targets even with intensive, multidrug therapy [5]. There is, however, observational evidence showing that even modest reductions in LDLC are associated with improved survival in patients with HoFH. In a retrospective observational study of HoFH patient from South Africa survival was improved in patients treated with statins compared to those who were treated before statins became available, despite a relatively modest response to statins [6]. Another study that combined data from South African and United Kingdom cohorts found that survival was best in the quartile of patients with the lowest on-treatment LDLC [7]. These findings were subsequently confirmed in a large international registry study (Homozygous Familial Hypercholesterolaemia International Clinical Collaboration - HICC) of patients with HoFH, supporting the concept of lowering LDLC as much as possible in patients with HoFH [5].

## Treatment of HoFH

Patients with HoFH require multiple therapies with different mechanisms of action to achieve LDLC control. In the HICC cohort only 2.6% of patients on a single therapy achieved their LDLC target compared to a 53.3% target achievement rate in those receiving five different therapies [5].

Surgical approaches to HoFH include liver transplantation, ileal bypass and portocaval shunt with the latter two operations no longer in use. Since not all LDL is cleared by the liver, transplantation in HoFH does not result in perfect control and may fail owing to rejection [8, 9]. The LDL reduction by ileal bypass relies on upregulation of functional receptors and consequently the response is usually poor. Portocaval shunt results in atrophy of the liver with reduced production of lipoproteins but may result in significant encephalopathy [10]. Apheresis started off as plasma exchange but has now largely been replaced by more selective LDL removal techniques [11–13].

Statins and ezetimibe form the backbone of conventional pharmacological lipid-lowering therapy and the current EAS guidelines recommend that high-dose statin + ezetimibe be started immediately upon diagnosis [1]. Because atorvastatin was found to lower LDLC significantly in all cases of HoFH in a placebo-controlled study that performed non-steady kinetic analysis after plasma exchange, statin therapy should be tried in all patients with HoFH. In the study LDLR null homozygotes had a 15% reduction in LDLC because the production of LDL was reduced, while LDL receptor defective homozygotes had better responses [14]. Ezetimibe lowered LDLC by 20.5% in HoFH patients receiving high-dose statin therapy, although an analysis by residual LDLR function is not available [15].

This review will discuss newer additional treatment options for HoFH according to molecular target and, if relevant, regulatory (FDA) approval date.

## Microsomal Triglyceride Transfer Protein (MTP)

Lomitapide is a small molecule drug that, when given orally, rapidly binds MTP in the lumen of the endoplasmic reticulum thereby inhibiting the lipidation and secretion of apoB-containing lipoproteins from both enterocytes (chylomicrons) and hepatocytes (VLDL). Lomitapide reduces LDLC by reducing VLDL production and less LDL is thus ultimately generated from intravascular modification of VLDL particles [16]. Lomitapide obtained marketing authorization from the FDA in December 2012 based on the results of a single-arm open-label forced titration study (5 mg daily to a maximum of 60 mg) of 29 adult patients in which lomitapide reduced LDLC by 50% at week 26. The effect of lomitapide was independent of the functional status (null/defective) of the participants LDLR pathogenic variants [17]. The LDLC reduction in a subsequent open-label extension study was − 45.5% in the 17 patients who completed week 126, despite some subjects discontinuing apheresis or increasing the intervals between apheresis treatments [18].

Subsequent real world observational studies have confirmed the efficacy of lomitapide in treating HoFH. In a study of 75 HoFH patients from 9 European countries the average LDLC reduction after a median of 19 months of treatment was 60% and the average lomitapide dose was 20 mg [19]. This LDLC reduction was observed despite 14 of the 38 patients who were receiving lipid apheresis at baseline discontinuing this treatment. In a retrospective comparison of an Italian cohort of patients treated with lomitapide and a French cohort treated with lipid apheresis the addition of lomitapide to conventional lipid-lowering therapy reduced LDLC by a further 58.0%, while LDLC was only reduced by a further 37.1% following initiation of lipid apheresis [20].

For many years the only pediatric data for lomitapide came from individual case reports and case series [21, 22]. In the largest of these case series, 11 patients under the age of 18 (mean age 11.6 ± 1.1 years) were treated with lomitapide (mean dose 24.5 ± 4.3 mg/day) and the mean observed LDLC nadir during treatment was 56.7% lower than baseline [21]. More recently in a single-arm open-label clinical trial of 43 pediatric patients the mean change in LDLC at week 24 was − 53.5% with no significant differences between participants aged 5–10 or 11–17 years [23].

The major adverse effects of lomitapide include gastrointestinal intolerance (mainly diarrhea), elevation of transaminases and hepatic steatosis [17]. Following registration of lomitapide, both the FDA and EMA required the manufacturer to establish an observational registry study of lomitapide to collect further efficacy and safety data [24]. The Lomitapide Observational Worldwide Evaluation Registry (LOWER) has confirmed the safety profile of lomitapide established in the registration and extension studies and has not identified any previously unknown risks [25]. A recent publication evaluated the long-term hepatic safety of lomitapide by aggregating data from multiple sources including the registration study and its extension, the LOWER registry and an Italian cohort study. The authors identified no cases of transaminitis accompanied by bilirubin elevation (Hy’s law) and in 34 Italian patients with sufficient data who had been treated with lomitapide for up to 9 years, elevations in hepatic fat were mild-to-moderate, hepatic stiffness as assessed by hepatic elastography remained normal, and the mean FIB-4 score did not increase significantly after a median exposure of 31.3 months (baseline 1.25 ± 1.05 versus 1.36 ± 1.12 at follow-up) [26].

### ApoB100

Mipomersen is a 20-base pair, 2’-O-methoxyethyl modified antisense oligonucleotide complementary to human apolipoprotein B-100 mRNA. Following subcutaneous injection it binds to apoB-100 mRNA in hepatocytes and the complex is then degraded by RNAses. Reduced production of apoB100 protein reduces the production of VLDL particles and thus ultimately LDL. In a study of 51 patients with HoFH mipomersen reduced LDLC by a mean of 24.7% in the 34 patients treated with mipomersen. LDLC reduction was highly variable ranging from + 2% to − 82%. Almost three quarters of patients treated with mipomersen experienced injection site reactions and transaminase elevations occurred in 12% [27]. In an open-label extension study similar LDLC reductions were maintained for up to 104 weeks, but many patients discontinued therapy because of side effects including injection site reactions, influenza-like symptoms post injection and transaminitis [28]. Mipomersen is also associated with the development of hepatic steatosis [29, 30]. Mipomersen was ultimately only approved by the FDA in January 2013 and not the EMA and is no longer commercially available.

### Proprotein Convertase Subtilisin/Kexin Type 9 (PCSK9)

Proprotein convertase subtilisin/kexin type 9 (PCSK9) is an enzymatically inactive serine protease which is predominantly secreted by hepatocytes [31]. PCSK9 binds to the LDLR on the cell surface and receptors with bound PCSK9 fail to recirculate to the cell surface following internalization, as PCSK9 prevents the dissociation of LDL from the LDLR resulting in lysosomal degradation of the LDLR [31]. Pathogenic variants in PCSK9 may either cause gain or loss of function, the former causes FH while the latter causes hypobetalipoproteinemia [32, 33]. Reduction of circulating PCSK9 (conventionally termed PCSK9 inhibition) increases the number of LDLR on the cell surface, resulting in increased clearance of LDL and thus lower LDLC concentrations.

Circulating PCSK9 can be reduced either by the administration of monoclonal antibodies binding PCSK9 (evolocumab or alirocumab) or by inhibiting its hepatic production using a siRNA (Inclisiran). Lerodalcibep is a fusion protein consisting of an adnectin based PCSK9 binding domain and human serum albumin and its mechanism of action is like that of the monoclonal antibodies [34].

### Monoclonal Antibodies

Alirocumab and evolocumab are fully human monoclonal antibodies directed against PCSK9. They reduce LDLC by approximately 50–60% in patients with polygenic hypercholesterolemia as well as HeFH when administered by subcutaneous injection either two-weekly or, in higher doses, monthly [35, 36]. In HeFH their efficacy is not affected by the type of LDLR pathogenic variant (null/defective) [36]. The first study to investigate the efficacy of these drugs in patients with HoFH was the TESLA Part A study. This was an open-label, single-arm, dose-scheduling pilot study in 8 patients with HoFH. All patients received evolocumab, initially dosed at 420 mg monthly for 12 weeks followed by 420 mg every two weeks for a further 12 weeks. Two participants were known to have LDLR null/null status and LDLC did not decrease in both these patients. In the six other patients LDLC reductions were 19.3 ± 16% and 26.3 ± 20% with the two-weekly and monthly dose, respectively [37]. In the larger TESLA Part B study, 50 patients aged 12 years or older with HoFH were randomized 2:1to either evolocumab 420 mg 4-weekly or placebo. Evolocumab reduced LDLC by 30.9% from baseline compared to placebo. The observed LDLC reduction gradated according to residual LDLR function and was − 46.9% in those with defective/defective status, −24.5% in those with defective/negative status, but LDLC increased by 10.3% in the single patient with confirmed negative/negative status [38]. However, responsiveness to evolocumab also varied markedly between patients with two copies of identical pathogenic variants and was shown to correlate with expression of LDLR on isolated lymphocytes [39].

The TAUSSIG study was an open-label extension study for participants from the TESLA studies, but also enrolled additional participants with HoFH and ultimately included 106 participants with HoFH. The mean reduction in LDLC at week 12 was 21.2% and in the 48 non-apheresis patients uptitrated to evolocumab 420 mg once every two weeks the reduction was − 19.6% at week 12 and − 29.7% after 12 weeks of treatment with the increased dose. However, the absolute LDLC reduction at week 48 (following the dose increase in the subgroup of patients) was only 2.06 mmol/L and given the mean baseline LDLC of 8.5 mmol/L most patients would require further therapy to reach their LDLC targets. LDLC reduction in patients on apheresis was 18.1% at week 12. Safety results in the 14 adolescent participants did not differ from adult patients [40].

In the Odyssey HoFH trial alirocumab dosed at 150 mg once every two weeks resulted in a mean difference in LDL-C percent change from baseline of −35.6% compared to placebo. Individual patient responses were once again highly variable – ranging from increases in LDLC to reductions approaching 80%. In contradistinction to the evolocumab studies where 105 of the 106 patients had a genetically confirmed HoFH diagnosis, 17 of the 69 participants in the Odyssey HoFH study did not have genetically confirmed HoFH. The commonest genetic abnormality in the 17 patients without genetically confirmed HoFH was a single pathogenic LDLR variant. LDLC reductions were on average larger in these patients than in those with bi-allelic pathogenic *LDLR* variants [41]. Most of the trials above were conducted predominantly in patients of Western origin. Studies conducted in other populations, with a different mix of pathogenic variants, have in some instances shown considerably lower mean responses. In a trial of 30 patients from India the mean LDLC reduction at week 12 was only 6.4% [42].

In an open-label, single arm pediatric study of 18 patients (age range 8–17 years, mean age 12.4 years) alirocumab administered at either 75 mg (body weight < 50 kg) or 150 mg (body weight > 50 kg) once every two weeks decreased LDLC by 4.1% at week 12. Half of the patients achieved LDLC reductions ≥ 15% at week 12. Responses were highly heterogenous and, on post-hoc genetic analysis, could at least partially be explained by 6 patients having *LDLR* null/null functional status and 2 patients having null/null mutations in the *LDLRAP1* gene [43]. In a pooled analysis of non-apheresis patients aged 10–17 years treated with evolocumab, week 12 median percentage change from baseline in LDL-C was − 2.9%; however, 42.9% (15/35) of patients achieved ≥ 15% reduction in LDL-C from baseline [44].

Lerodalcibep is given by subcutaneous injection once a month. It has been evaluated in HoFH patients aged > 10 years in a crossover study in which participants were initially randomized to either 24 weeks of evolocumab 420 mg once every 4 weeks or lerodalcibep 300 mg once every 4 weeks for 24 weeks (NCT04034485). Patients then crossed over to the alternative treatment for another 24-week period following an 8-week washout. The results of this study have thus far only been published in abstract form. The study enrolled 65 patients of whom 56 completed both treatment arms. Mean reduction in LDLC was − 9.6% with lerodalcibep and − 11.7% with evolocumab. LDLC reductions were once again highly variable but were similar with both products in individual participants. (*r* = 0.79; *p* < 0.001) [45].

Given that alirocumab and evolocumab, in both short-term and long-term studies (up to 8.4 years exposure for evolocumab), have excellent safety profiles that do not differ from placebo in most respects, except for a higher incidence of injection site reactions [46], the current EAS treatment guidelines recommend that treatment with monoclonal antibodies directed against PCSK9 be initiated within 8 weeks of starting therapy with a statin and ezetimibe if the patient is not at LDLC target, except for patients with known null/null functional receptors. The response should be assessed following 1–2 doses and treatment with PCSK9-inhibition discontinued in those where the additional LDLC reduction is < 15% [1].

### Inclisiran

Inclisiran is a hepatocyte-directed siRNA directed against the RNA for production of PCSK9. It is given by subcutaneous injection once every 6 months following initial dosing at months 0 and 3. It has shown good efficacy in the treatment of HeFH with a mean placebo-corrected LDLC reduction from baseline of 47.9% [47]. In a small pilot study of 4 HoFH patients PCSK9 was reduced markedly in all 4 participants, but LDLC was reduced by − 11.7% to − 33.1% at day 90 in only three participants while the fourth participant showed no response [48]. Following the pilot study a large, double-blind, placebo-controlled trial was initiated. In this trial 56 HoFH patients were randomized in a 2:1 ratio to either Inclisiran sodium 300 mg or placebo. PCSK9 levels were reduced by 60.6% with Inclisiran but the placebo corrected change in LDLC was not statistically significant at −1.68%. Once again LDLC changes were highly heterogenous with large increases or reductions in some patients [49]. There are multiple possible explanations for the failure of Inclisiran to lower LDLC in this study including a high production rate of PCSK9 in patients with HoFH, an overrepresentation of null/null patients in the Inclisiran group, the inclusion of patients who had previously not responded to monoclonal antibodies, and the inclusion of patients on apheresis as changes in apheresis timing may influence LDLC levels markedly.

### Angiopoietin-like protein 3 (ANGPTL3)

Angiopoietin-like protein 3 (ANGPTL3), ANGPTL4 and ANGPTL8 are important regulators of lipoprotein metabolism and play an important role in regulating triglyceride-rich lipoprotein metabolism depending on whether the body is in a fasted or fed state. ANGPTL3 is predominately synthesized in the liver. ANGPTL3 secretion is regulated by liver X receptors (LXR) and hepatocyte nuclear factor 1 (HNF-1). ANGPTL3 forms a complex with ANGPTL8 and this complex exerts a strong inhibitory activity on lipoprotein lipase (LPL) as well as endothelial lipase (EL) [50–52].

Loss of function variants in *ANGPTL3* are associated with familial combined hypolipidemia which is characterized by a reduction in all circulating lipoproteins [53]. There is a gene-dose effect and those with bi-allelic variants have more severe hypolipidemia, but no other clinically overt pathology [54]. Loss of function mutations in *ANGPTL3* also correlate with decreased risk of ASCVD in epidemiological studies, while inhibition of ANGPTL3 in atherosclerosis-prone mice with the fully human IgG4 monoclonal antibody evinacumab was associated with reductions in atherosclerotic lesions [55].

Evinacumab is thought to reduce LDLC in HoFH by activation of LPL and EL. This results in enhanced lipolysis with increased production of smaller VLDL-remnants and IDL particles, uptake of these particles via LDLR-independent mechanisms and less generation of LDL in the circulation [56].

In a double-blind, randomized, placebo-controlled trial, 65 patients with HoFH on stable lipid-lowering therapy including apheresis, were assigned in a 2:1 ratio to either receive evinacumab (15 mg/kg by intravenous infusion over 1 h monthly) or placebo. At week 24 placebo-corrected LDLC was reduced by 49% and, importantly, there was no difference in response between those with null/null functional LDLR status or non-null status [57].

Evinacumab was subsequently further evaluated in a long-term open-label study. This study included patients who had participated in previous evinacumab studies as well as evinacumab-naïve patients. In total the study enrolled 116 patients (14 adolescents aged 12 to 18 years). The median duration of study participation was 104 weeks. Evinacumab reduced LDLC by a mean value of 43.6% and these reductions were maintained for up to 120 weeks of treatment. Once again LDLR functional status did not influence the response. Evinacumab was well tolerated, and no significant safety concerns were identified [58].

In a pediatric open-label study 14 patients (aged 5–11 years) received evinacumab 15 mg/kg by monthly infusion. The mean LDLC reduction at week 24 was 48.3%. In a pharmacokinetic substudy the measured serum evinacumab concentrations were within the parameters predicted by a pharmacokinetic model and did not differ from those in adults. Lipoprotein apheresis reduced evinacumab concentration by 10–25%. Evinacumab was well tolerated in this small study and no new safety signals were identified [59]. Another monoclonal antibody directed against ANGPTL3 (SHR-1918) is set to enter clinical trials soon (NCT06009393) [60].

ANGPTL3 concentrations can either be reduced with monoclonal antibodies or by specific inhibition of protein synthesis using RNA therapeutics. Vupanorsen is an antisense oligonucleotide (ASO) directed against ANGPTL3 and could potentially be effective in patients with HoFH. Further development of vupanorsen was, however, discontinued following the results of the TRANSLATE trial in which increasing doses of vupanorsen were administered to statin-treated patients with residual hyperlipidemia. Plasma ANGPTL3 was reduced by up to 95% with the highest doses, but the proportion of patients with hepatic transaminase elevations increased with higher doses and was 44.4% in patients dosed with 160 mg biweekly. Similarly, the hepatic fat fraction increased in the higher dose groups with an increase of up to 76% in the highest dose group [61].

Zodasiran (formerly ARO-ANG3) is a siRNA directed against ANGPTL3. Its effect in patients with HoFH was evaluated in an open-label study of 18 patients with HoFH who were dosed with either 200 mg or 300 mg of Zodasiran on day 1 and at week 12. At week 20 mean absolute reduction in LDLC from baseline were − 4.4 mmol/L and − 5.2 mmol/L for the 200 mg and 300 mg doses respectively corresponding to −48.1% and − 43.9% mean reductions. Zodasiran was generally well tolerated in this short and small study, although hepatic fat fraction was not assessed systematically [62]. However, in other studies Zodasiran has not been associated with increases in hepatic fat fraction [63]. Evinacumab has also not been associated with increases in hepatic fat and when administered to patients with elevated triglycerides was associated with a 16% relative decrease in hepatic fat fraction [64]. It is difficult to explain these differences in the risk of developing hepatic steatosis between the various agents inhibiting ANGPTL3, particularly between the two that both decrease hepatic ANGPTL3 synthesis. One potential explanation may relate to the degree to which ANGPTL3 synthesis is inhibited. Changes in hepatic fat fraction and transaminases were dose related with vupanorsen and only seen with the highest doses. These doses reduced circulating ANGPTL3 by up to 95%, more than the 65–85% reductions seen with Zodasiran. Very potent inhibition of ANGPTL3 production may reduce the secretion of apoB-containing lipoproteins from the liver, leading to hepatic fat accumulation [65].

The manufacturer of Zodasiran recently announced the cancellation of a planned phase 3 trial in patients with HoFH for commercial reasons [66]. Two other siRNA therapies targeting ANGPTL3 (solbinsiran and ALN-ANG3) have not yet been trialed in patients with HoFH.

### DNA Based Therapies

While RNA-targeted therapies usually aim to reduce gene expression, DNA-targeted therapies have traditionally aimed to introduce normal functional copies of the defective gene. Pathogenic variants in the *LDLR* gene are responsible for most cases of HoFH and Grossman and colleagues published the results of a small trial in 1995 in which 5 patients were treated with ex-vivo gene therapy - resected hepatocytes were transfected ex-vivo with *LDLR* cDNA using a murine retroviral vector and subsequently infused into the portal vein. Reductions in LDLC were demonstrated in 3 of the 5 patients, but transgene expression was only detected in a relatively small number of hepatocytes [67, 68]. Given the invasive nature of this technique, the relatively low efficiency of transfection, the uncertainty about the long-term persistence of expression of normal LDL receptors and concerns regarding long-term safety of retroviral vectors this approach was not pursued further. Subsequent attempts at gene therapy used adeno-associated virus 8 (AAV8), but no clinically relevant reductions in LDLC were seen in the 9 patients enrolled and there was an elevation in transaminases [69]. An alternative approach to using viral vectors has been proposed, delivering *LDLR* mRNA to hepatocytes using exosome technology (NCT05043181). No results from this trial have been published yet [70].

The approaches described above can be described as gene replacement therapy as they aim to replace the defective gene with a wild-type copy. The discovery of the clustered-regularly interspaced-short-palindromic-repeat/CRISPR-associated gene 9 (CRISPR–Cas9) system has allowed gene-editing approaches – either repairing the defective gene or permanently affecting the function of important regulatory genes such as *PCSK9* or *ANGPLT3*. Verve-101 and Verve-102 are gene-editors directed against PCSK9 while Verve-201 is a ANGPTL-3-directed base editor targeted to hepatocytes using GalNAc-conjugated lipid nanoparticles. As yet, these agents have not yet been used in patients with HoFH but Verve-101, directed against PCSK9, dosed at 0.45 mg/kg cohort in five participants with follow-up to at least 28 days demonstrated time-averaged LDL-C reductions ranging from 21 to 73% and averaging 46% [71]. For HoFH, gene-editing of ANGPTL3 is likely to be more effective as, unlike PCSK9 editing, it is independent of LDLR function.

## Conclusions

The last few years have seen major advances in the treatment of patients with HoFH. Before effective therapies for HoFH became available survival beyond early adulthood was uncommon. Despite only being modestly effective, statin-based therapy was already associated with improved survival with subsequent studies confirming that survival improves progressively with LDLC control. In a modelling analysis several strategies for HoFH treatment were analyzed, starting with no treatment followed by high-intensity statin treatment, high-intensity statins and ezetimibe, high-intensity statins + ezetimibe + PCSK9 inhibitors and finally high-intensity statins + ezetimibe + PCSK9 inhibitors + evinacumab. In the first model the hazard ratio for cardiovascular mortality for a 1 mmol/L reduction in LDLC was estimated to be 0.77 (model 2 assumed a hazard ratio of 0.88) and both models assumed continuous, lifelong therapy with perfect adherence. The median life expectancy for model 1 was 33 years. Adding high-intensity statin therapy increased survival by 9 years and adding ezetimibe to statins added a further 9 years. The addition of PCSK9 directed therapy increased life expectancy by a further median of 14 years, while addition of evinacumab to all previous therapies added a further 12 years of life. In sensitivity analyses assuming different baseline LDLC values and a HR 0f 0.88 per mmol/L reduction the effect of adding evinacumab varied between adding 7–12 years of life expectancy [72]. The effect of lomitapide was not assessed in this model, but since LDLC reductions with lomitapide are like those achieved with evinacumab, and independent of residual LDLR function, it is reasonable to assume similar effects on survival as long as there in no competing mortality from the effects on lomitapide on the liver.

The management of HoFH thus relies on early identification and diagnosis and initiation of therapy as soon as possible with rapid addition of additional therapies to bring patients as close as possible to their LDL-C target. However, many therapies lack data for use in the very young. The current EAS guidelines recommend starting with a statin + ezetimibe combination and then rapidly adding PCSK9 directed therapy. The PCSK9 directed therapy should be continued if it reduces LDLC by a further15% or greater [1]. Further therapies will be required for most patients and in clinical practice the choice of therapy will often be directed by reimbursement and availability. Further pharmacological therapies could include the LDLR independent therapies, lomitapide or evinacumab (or both) while additional non-pharmacological therapies could be lipid apheresis or hepatic transplantation. Gene editing to inactivate *ANGPTL3* may also benefit patients with HoFH in the future.

In addition to focusing on LDLC reduction further ancillary approaches requiring evaluation may include control of Lp(a) with RNA-targeting therapies in those HoFH patients with elevated Lp(a) levels, anti-inflammatory therapies such as colchicine, and the use of EPA supplements or HDL-mimetic therapies.

Worldwide the largest barrier to optimal care of patients with HoFH is the limited access to novel, LDLR-independent therapies. Currently, these therapies are very costly and are not available in many countries, particularly lower and middle-income countries, leading to poor LDLC control and increased mortality in these high-risk patients [5].

## Key Rerefences

- Cuchel M, Raal FJ, Hegele RA, Al-Rasadi K, Arca M, Averna M, et al. 2023 Update on European Atherosclerosis Society Consensus Statement on Homozygous Familial Hypercholesterolaemia: new treatments and clinical guidance. European heart journal. 2023;44(25):2277-91. 10.1093/eurheartj/ehad197. Most recent consensus statement on the management of HoFH
- Tromp TR, Hartgers ML, Hovingh GK, Vallejo-Vaz AJ, Ray KK, Soran H, et al. Worldwide experience of homozygous familial hypercholesterolaemia: retrospective cohort study. Lancet. 2022. 10.1016/S0140-6736(21)02001-8. The largest and most recent worldwide cohort study of patients with HoFH.
- Raal FJ, Pilcher GJ, Panz VR, van Deventer HE, Brice BC, Blom DJ, et al. Reduction in mortality in subjects with homozygous familial hypercholesterolemia associated with advances in lipid-lowering therapy. Circulation. 2011;124(20):2202-7. 10.1161/CIRCULATIONAHA.111.042523. The first study to show that statin therapy was associated with improved outcomes in patients with HoFH.
- Thompson GR, Blom DJ, Marais AD, Seed M, Pilcher GJ, Raal FJ. Survival in homozygous familial hypercholesterolaemia is determined by the on-treatment level of serum cholesterol. European heart journal. 2018;39(14):1162-8. 10.1093/eurheartj/ehx317. This study showed that survival was best in patients with the lowest LDLC.
- Cuchel M, Meagher EA, du Toit Theron H, Blom DJ, Marais AD, Hegele RA, et al. Efficacy and safety of a microsomal triglyceride transfer protein inhibitor in patients with homozygous familial hypercholesterolaemia: a single-arm, open-label, phase 3 study. Lancet. 2013;381(9860):40-6.10.1016/S0140-6736(12)61731-0.This is the pivotal study that led to the registration of lomitapide.
- D'Erasmo L, Steward K, Cefalù AB, Di Costanzo A, Boersma E, Bini S, et al. Efficacy and safety of lomitapide in homozygous familial hypercholesterolaemia: the pan-European retrospective observational study. Eur J Prev Cardiol. 2022;29(5):832-41. 10.1093/eurjpc/zwab229. The largest cohort study of lomitapide published thus far.
- Larrey D, D'Erasmo L, O'Brien S, Arca M, Italian Working Group on L. Long-term hepatic safety of lomitapide in homozygous familial hypercholesterolaemia. Liver Int. 2023;43(2):413-23. 10.1111/liv.15497.This study summarizes hepatic safety data for lomitapide from multiple cohorts.
- Raal FJ, Santos RD, Blom DJ, Marais AD, Charng MJ, Cromwell WC, et al. Mipomersen, an apolipoprotein B synthesis inhibitor, for lowering of LDL cholesterol concentrations in patients with homozygous familial hypercholesterolaemia: a randomised, double-blind, placebo-controlled trial. Lancet. 2010;375(9719):998-1006. 10.1016/S0140-6736(10)60284-X. The pivotal study of mipomersen in HoFH.
- Stein EA, Honarpour N, Wasserman SM, Xu F, Scott R, Raal FJ. Effect of the proprotein convertase subtilisin/kexin 9 monoclonal antibody, AMG 145, in homozygous familial hypercholesterolemia. Circulation. 2013;128(19):2113-20. 10.1161/CIRCULATIONAHA.113.004678. The first trial to evaluate the use of PCSK9 inhibitors in patients with HoFH.
- Raal FJ, Honarpour N, Blom DJ, Hovingh GK, Xu F, Scott R, et al. Inhibition of PCSK9 with evolocumab in homozygous familial hypercholesterolaemia (TESLA Part B): a randomised, double-blind, placebo-controlled trial. Lancet. 2015;385(9965):341-50. 10.1016/S0140-6736(14)61374-X. The first large study of a PCSK9 inhibitor in HoFH.
- Raal F, Durst R, Bi R, Talloczy Z, Maheux P, Lesogor A, et al. Efficacy, Safety, and Tolerability of Inclisiran in Patients With Homozygous Familial Hypercholesterolemia: Results From the ORION-5 Randomized Clinical Trial. Circulation. 2024;149(5):354-62. 10.1161/CIRCULATIONAHA.122.063460. The pivotal study of inclisiran in HoFH.
- Raal FJ, Rosenson RS, Reeskamp LF, Hovingh GK, Kastelein JJP, Rubba P, et al. Evinacumab for Homozygous Familial Hypercholesterolemia. The New England journal of medicine. 2020;383(8):711-20. 10.1056/NEJMoa2004215. The pivotal trial of evinacumab in HoFH.

## Data Availability

No datasets were generated or analysed during the current study.
